# Constructing MoO_2_ Porous Architectures Using Graphene Oxide Flexible Supports for Lithium Ion Battery Anodes

**DOI:** 10.1002/gch2.201700050

**Published:** 2017-08-28

**Authors:** Zhanwei Xu, Kai Yao, Hao Fu, Xuetao Shen, Xintong Duan, Liyun Cao, Jianfeng Huang, Huanlei Wang

**Affiliations:** ^1^ School of Materials Science and Engineering Shaanxi University of Science and Technology Xi'an 710021 China; ^2^ Institute of Materials Science and Engineering Ocean University of China Qingdao 266100 China

**Keywords:** graphene oxide, lithium battery anode, molybdenum dioxide, nanoarchitecture, preform

## Abstract

Graphene oxide flexibly supported MoO_2_ porous architectures (MoO_2_/GO) by decomposition of the prepared ammonium molybdate/GO preforms is fabricated. Focused ion beam microscope analysis shows that the inside structures of the architectures strongly depend on the percentages of the GO used as flexible supports: micrometer scale MoO_2_ particulates growing on the GO (micrometer MoO_2_/GO), 3D honeycomb‐like nanoarchitectures (MoO_2_/GO nanohoneycomb), and layered MoO_2_/GO architectures are achieved at the percentage of GO at 4.3, 15.2, and 20.8 wt%, respectively. The lithium storage performance of the MoO_2_/GO architectures strongly depends on their inside structures. At the current density of 100 mA g^−1^, the capacities of the micrometer MoO_2_/GO, MoO_2_/GO nanohoneycomb, and layered MoO_2_/GO remain at 901, 1127, and 967 mAh g^−1^ after 100 cycles. The average coulombic efficiencies of micrometer MoO_2_/GO, MoO_2_/GO nanohoneycomb, and layered MoO_2_/GO electrodes are 97.6%, 99.3%, and 99.0%. Moreover, the rate performance shows even cycled at a high current density of 5000 mA g^−1^, the MoO_2_/GO nanohoneycomb can deliver the capacity as high as 461 mAh g^−1^. The MoO_2_/GO nanohoneycomb exhibits best performance attributed to its unique nanohoneycomb structure constructed with ultrafine MoO_2_ fixed on the GO flexible supports.

## 1 Introduction

Lithium ion batteries (LIBs) have attracted much interest due to their many applications in the fields of electric vehicles and portable electronic devices, such as mobile phones, laptop computers, and video cameras. Nowadays graphite is employed as the dominant anode materials for commercial LIBs. However, the theoretical capacity of graphite electrodes is 372 mAh g^−1^, resulted in an obstacle for their potential applications. The strategies to develop alternative anode materials with improved capacity have been achieved for several decades.^1^, ^2^, ^3^, ^4^, ^5^, ^6^, ^7^, ^8^ Among them, nanoscale metal oxides, including MnO,^9^ SnO_2_,^10^, ^11^ Co_3_O_4_,^12^ NiO,^13^ and Fe_3_O_4_,^14^, ^15^ used as anode active materials for LIBs have been paid much attention attributed to their promising theoretical capacity. However, in most cases, the capacity decays rapidly as cycled due to high electrical resistivity and mechanism failure.^16^ Molybdenum oxide (MoO_2_) is different from other metal oxides. It possesses metallic conductivity (its electrical resistivity is 8.8 × 10^−5^ Ω cm at 300 K in bulk sample), with a theoretical capacity as high as 836 mAh g^−1^.^17^ However, the capacity of bulk MoO_2_ is low due to sluggish lithiation/delithiation kinetics.^17^, ^18^, ^19^, ^20^

On the other hand, the nanoporous structure constructed with lots of interconnect nanounits to form numbers of nanoscale pore provides adequate spaces for electrochemical reaction and connected pathways for ion diffusion.^21^, ^22^ The nano‐MoO_2_ porous structures have been synthesized by using mesoporous silica as hard templates, followed removing this hard templates by hydrofluoric acid,^23^ or by a sulfur‐assisted decomposition process.^24^ The prepared MoO_2_ porous materials have displayed improved capacity for LIBs. However, the capacity fade after charge/discharge process is attributed to the pure MoO_2_ skeleton that will be possibly collapsed. The cycle stability needs to be improved further. It should be desired for the MoO_2_ porous structure with flexible supports via retarding the pulverization from pure MoO_2_ skeleton.

Carbon nanomaterials, especially, graphene with large specific surface area and high toughness, are suitable for both substrates and supports for MoO_2_ electrode active materials. However, owing to graphene being liable to stack together, it is difficult to prepare well distributed graphene templated metal oxide architectures. The strategies, including solid‐state graphenothermal reduction method,^25^ microwave‐assisted hydrothermal process,^26^ and soft‐templated hydrothermal method,^27^ have been used to synthesize MoO_2_/C nanocomposites, exhibiting promising performance for LIBs. In our present study, in order to construct the MoO_2_‐based nanoporous which can baffle the mechanical failure, the graphene oxide (GO) was used as the flexible supports for MoO_2_ active material to slow down the stress during charge/discharge process and keep the structure intact. The GO supported MoO_2_ nanoarchitectures were fabricated by a preform ammonium molybdate/GO prepared, followed a solid decomposition process, ensuring the GO uniform dispersion and to construct well‐distributed porous materials.^28^ Focused ion beam (FIB) microscope analysis was employed to show the inside structure, which is the main part as the porous materials. It shows the quantity of the GO supports strongly affects the inside structures of the MoO_2_/GO architectures: micrometer scale MoO_2_ particles growing on the graphene oxide (micrometer MoO_2_/GO), 3D honeycomb‐like nanoarchitecture (MoO_2_/GO nanohoneycomb), and MoO_2_/GO layered structure (layered MoO_2_/GO) are achieved at the percentage of graphene 4.3, 15.2, and 20.8 wt%, respectively. The analysis based on the galvanostatic discharge/charge voltage profiles, differential curves, cycle life behavior as well as rate performance demonstrates their lithium storage performance strongly depend on the inside structures of the MoO_2_/GO architectures.

## 2 Results and Discussion

**Scheme 1:**
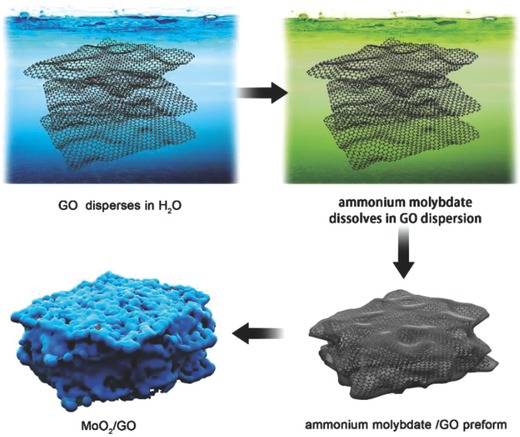
Schematic illustration of MoO_2_/GO synthesis and structure. The MoO_2_/GO is prepared by decomposition of an ammonium molybdate/GO preform.

In order to show the effects of the inside pore structure of MoO_2_/GO on the performance used as LIB anodes, the GO used as flexible supports and a ammonium molybdate were adjusted on various ratios to build up kinds of pore structures of MoO_2_/GO. A preform‐decomposition process was employed to synthesize MoO_2_/GO architectures,^28^ which are shown in **Scheme** **1**. The GO disperses in water, then ammonium molybdate dissolves in GO aqueous dispersion. After evaporation of water, an ammonium molybdate/GO preform formed. The MoO_2_/GO architectures were achieved by decomposition of the preform at around 500 °C under the atmosphere of argon.

**Figure 1:**
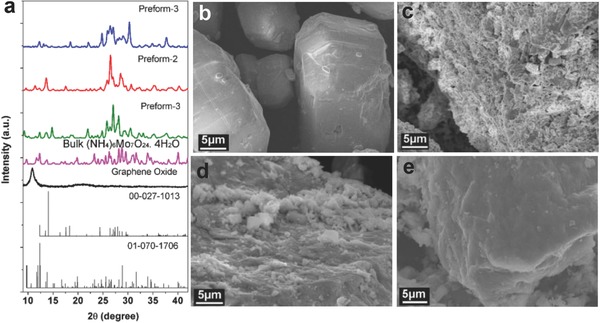
a) XRD patterns of the prepared preform‐1–3, (NH_4_)_6_Mo_7_O_24_·4H_2_O, and graphene oxide. SEM images of b) the bulk (NH_4_)_6_Mo_7_O_24_·4H_2_O, c) the prepared preform‐1, d) preform‐2, and e) preform‐3.

**Figure** **1**a shows the X‐ray diffraction (XRD) patterns of the GO, bulk (NH_4_)_6_Mo_7_O_24_·4H_2_O, and the prepared preforms, in which the weight ratios of ammonium molybdate: GO are 20:1, 10:1, and 5:1. The XRD patterns of the GO supports show the peak around 2θ = 9.8°, indicating interlayer spacing is 0.80 nm. Another peak at 2θ = 24.0° (0.37 nm) has appeared, suggesting a trace of graphite flakes present in the GO.^29^ XRD patterns show the prepared preforms are composed of (NH_4_)_2_Mo_2_O_7_ mainly (ICDD 00‐027‐1013), a small amount of (NH_4_)_6_Mo_7_O_24_·4H_2_O (ICDD 01‐070‐1706) as well as the GO. Scanning electron microscopy (SEM) image of the raw material (NH_4_)_6_Mo_7_O_24_·4H_2_O displays a large particle bulk morphology (Figure 1b). SEM image s of the prepared preforms‐1–3 are shown in Figure 1c–e, respectively. A loose layered structure composed of a great number pieces of (NH_4_)_2_Mo_2_O_7_ or (NH_4_)_6_Mo_7_O_24_·4H_2_O are obtained at the low ratio of GO supports (the weight ratio is 1:20, Preform‐1, Figure 1c), and a very dense layered structure is prepared at the high ratio of GO supports (the weight ratio is 1:5, Preform‐3, Figure 1e). At a moderate percentage GO, it can be observed from the cross‐section the prepared preform shows a transmit morphology among them (the weight ratio is 1:10, Preform‐2, Figure 1d).

**Figure 2:**
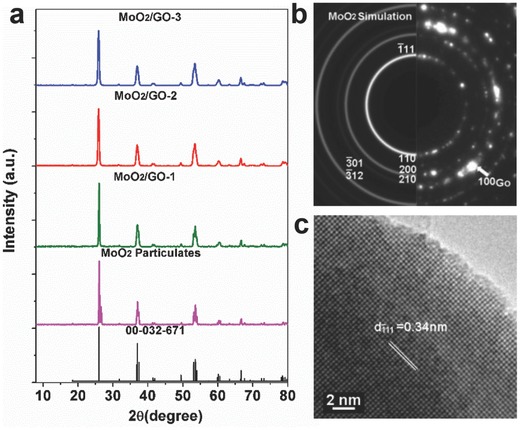
a) XRD patterns of the MoO_2_/GO architectures and the reference MoO_2_. b) Typical SAED patterns of MoO_2_/GO‐2. c) High‐resolution transmission electron microscopy (HRTEM) image of the MoO_2_.

**Figure** **2**a shows the XRD patterns of the MoO_2_/GO‐1–3 prepared by decomposition of the preform‐1–preform‐3 as well as the reference MoO_2_ particulates synthesized by the decomposition of bulk (NH_4_)_6_Mo_7_O_24_·4H_2_O (SEM morphology of the MoO_2_ particles are shown in Figure S1, Supporting Information). All of the diffraction peaks of the samples and the reference can be indexed to MoO_2_ (ICDD 00‐032‐671, P21 (4), *a* = 5.610, *b* = 4.843, *c* = 5.526).^30^ The reference MoO_2_ particles and the MoO_2_/GO‐1 display 2 peaks in the range 26.00°, assigned the reflections of (110) and (011), two peaks at 37.00° and 37.30°, assigned to the reflections of (020) and (111), as well as three peaks at 53.10°, 53.50°, and 53.90°, assigned to the reflections of (−222), (022), and (112). As to the MoO_2_/GO‐2, and MoO_2_/GO‐3, the peaks around 26.00°, 37.20°, and 53.50° display three broaden and merged peaks, indicating that the size of MoO_2_ of MoO_2_/GO‐2, MoO_2_/GO‐3 is smaller than the reference MoO_2_ particles as well as the MoO_2_/GO‐1, which will be confirmed further by FIB–SEM analysis. Different from the preforms, the XRD pattern related to the well‐distributed GO can hardly to be observed, attributed to the MoO_2_ growth on GO surface, and the stacking of the graphene was inhibited.^31^ Transmission electron microscopy (TEM) spectroscopy analysis was employed to get more information. For TEM observation, the sample MoO_2_/GO‐2 was disperse in ethanol. After a strong ultrasonic vibration, the MoO_2_ nanoparticles and the GO were exposed. The selected area electron diffraction (SAED) patterns indicate the MoO_2_ particle is a nanocrystalline phase (Figure 2b). The MoO_2_ has highly crystallized structure with the interplanar distance of 0.34 nm, corresponding to the d‐spacing of its (−111) reflection (Figure 2c).

The average atomic ratios of the products were determined by energy dispersive X‐ray spectroscopy (EDS) analysis performed with a Hitachi S‐3000 N scanning electron microscope (shown in **Table** **1**). It shows the ratios of MoO_2_:C in the MoO_2_/GO‐1–3 are 95.7:4.3, 84.8:15.2, and 79.2:20.8 by weight. The atomic ratios of Mo, C, and O elements are also provided in Table 1. The ratios of O: Mo is higher than the value 2 possible attributed that a small amount of MoO_2_ in the surface oxidized in air or from the graphene‐oxide supports.^30^ X‐ray photoelectron spectroscopy (XPS) spectra show the surface atomic composition of Mo, C, and O of MoO_2_/GO‐1 are 26.5%, 19.2%, and 54.3%, respectively. The surface of MoO_2_/GO‐2 is composed of 20.43%, 36.57%, and 43.00% of Mo, C, and O atoms, respectively. While, the MoO_2_/GO‐3 is composed of 14.48, 53.02, and 32.51% of Mo, C, and O atoms at surface. The results are consistent with the EDS analysis.

**Table 1:** Elemental composition of GO substrated MoO_2_

As prepared composite	wt%			at%		
	C	O	Mo	C	O	Mo
MoO_2_/GO‐1	4.41	24.62	70.99	23.29	52.40	24.31
MoO_2_/GO‐2	15.24	21.82	62.94	38.58	41.47	19.95
MoO_2_/GO‐3	21.58	21.09	57.33	48.52	35.56	16.11

**Figure 3:**
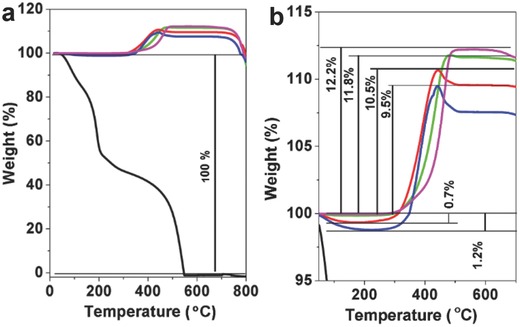
a) TGA curves of the MoO_2_/GO architectures, MoO_2_/GO‐1 (green), MoO_2_/GO‐2(red), MoO_2_/GO‐3(blue), the reference MoO_2_ particulates (pink), and graphene oxide (black). b) Enlarged TGA curves from (a).

The air thermal gravimetric analysis (TGA) curve of the baseline MoO_2_ particles shows two main weight change steps. The first is a significant weight increase starting at around 410 °C and lasting to ≈500 °C. This is attributed to the oxidation of MoO_2_ to MoO_3_, with a corresponding 12.2% weight increase that agrees well with theoretical value 12.5%. The second step is a large weight loss starting at around 760 °C due to the sublimation of MoO_3_.^24^ The air TGA curve of GO exhibits two weight loss steps. The initial weight loss occurs starting at around 50 °C and lasting to around 150 °C, attributed to the evaporation of physically adsorbed water on the higher surface area GO.^21^ The exothermal peak at ≈510 °C can be assigned to the ignition of GO. As to the MoO_2_/GO‐1, MoO_2_/GO‐2, and MoO_2_/GO‐3, the first is a significant weight increase starting at around 400, 330, and 350 °C, respectively. The MoO_2_/GO‐2 exhibits the earliest, followed by MoO_2_/GO‐3, then MoO_2_/GO‐1, possibly attributed that the MoO_2_ with nanosize is liable to oxide into MoO_3_ in air than micrometer or bulk MoO_2_. The TGA curves of the MoO_2_/GO‐2, MoO_2_/GO‐3 achieved their peaks around 400 °C, then dropped. As to MoO_2_/GO‐1, a weak peak around 420 °C occurred. However, there is no peak, just a platform appeared in the TGA curve of the pure bulk MoO_2_ (**Figure** **3**). These are attributed to the oxidation of MoO_2_ of the MoO_2_/GO architectures to MoO_3_ and the ignition of GO of MoO_2_/GO happened simultaneously during this period. There is a higher quantity of GO in the MoO_2_/GO‐2, MoO_2_/GO‐3 than MoO_2_/GO‐1. To the MoO_2_/GO‐1, MoO_2_/GO‐2, and MoO_2_/GO‐3, the corresponding weight increases 11.8%, 10.5%, and 9.5%, respectively. These values are consistent to the EDS analysis.

**Figure 4:**
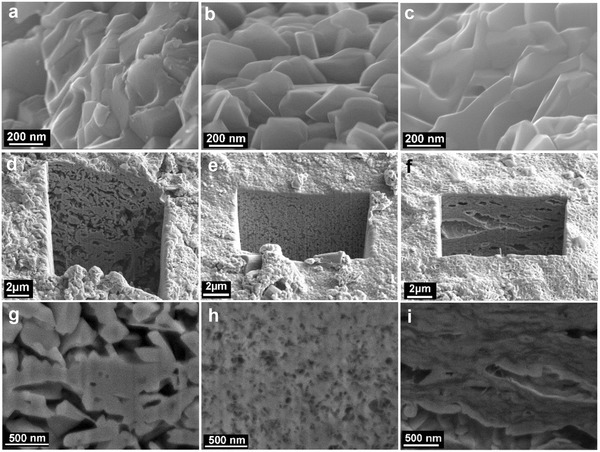
Field emission scanning electron microscopy (FESEM) images of a) MoO_2_/GO‐1, b) MoO_2_/GO‐2, and c) MoO_2_/GO‐3. FIB images of d) MoO_2_/GO‐1, e) MoO_2_/GO‐2, and f) MoO_2_/GO‐3. Magnified FIB images of g) MoO_2_/GO‐1, h) MoO_2_/GO‐2, and i) MoO_2_/GO‐3.

SEM analysis and FIB–SEM analysis were employed to show the morphologies both the surface and inside of the prepared MoO_2_/GO composites (**Figure** **4**). From the views of the surface SEM images, very dense MoO_2_ nanoparticles around 200 nm in size connected each other can be observed in all of the graphene‐oxide supported MoO_2_ assemblies. (Figure 4a–c). It is very difficult to distinguish the composites with various ratios of MoO_2_ to GO based on their surface morphologies. FIB–SEM analysis was employed to show the inside structures of the porous materials. A composite of micrometer (around 0.5 μm) MoO_2_ particles growing on the graphene oxide with irregular micrometer level connected hollows (from 0.2 to 1 μm) is obtained at the low ratio of GO (4.3% GO by weight, micrometer MoO_2_/GO) (Figure 4d,g). A hybrid with MoO_2_ thinner layer‐graphene‐oxide layered structure is obtained at the high ratio of GO (20.8% GO by weight) (Figure 4f,i), mingled with irregular microchannels (layered MoO_2_/GO). The inside of the architectures exhibits sandwich like structure both at higher and lower ratios of MoO_2_ to GO. However, when we control the ratio at optimized value (MoO_2_ to GO is 84.8:15.2 wt%), a 3D honeycomb‐like structure with nanoscale pores around 50 nm in size is achieved (MoO_2_/GO nanohoneycomb, Figure 4e,h). TEM microscopy was employed to investigate the structures of MoO_2_/GO architectures further. The TEM image of micrometer MoO_2_/GO displays MoO_2_ in the size around 0.5 μm connected to GO (provided in Figure S3a, Supporting Information). By contrast, the MoO_2_/GO nanohoneycomb exhibits GO substrates anchored by lots of nanosize (≈100 nm) MoO_2_ (Figure S3b, Supporting Information). As for the layered MoO_2_/GO, the TEM shows MoO_2_ sheets covered on GO substrates (Figure S3c, Supporting Information). These are consistent with the FIB–SEM analysis. The results indicate the ratios of GO supports strongly affect the inside structure of MoO_2_ assemblies. Nitrogen adsorption–desorption isotherms of MoO_2_/GO architectures are provided in Figure S4 in the Supporting Information. The surface area of the micrometer MoO_2_/GO, MoO_2_/GO nanohoneycomb and layered MoO_2_/GO are 9.3, 38.4 and 11.2 m^2^ g^−1^, respectively. The MoO_2_/GO nanohoneycomb exhibits the highest surface area among them. Fourier transform infrared spectroscopy (FTIR) spectrum shows the presence of C=O (1716 cm^−1^), C—O (1015 cm^−1^), OH (3361 cm^−1^), and COOH (3583 cm^−1^) in the GO (Figure S5a, Supporting Information). These functional groups make the GO connect with MoO_2_ via hydrogen bond between H of GO and O of MoO_2_ as well as the coordination bond through the O of GO and Mo of MoO_2_ (Figure S5b, Supporting Information). The MoO_2_/GO‐2 has high surface area. A large interface and strong force exist between the nanoscale MoO_2_ and the GO. Therefore, the GO connected with nanoscale MoO_2_ tightly, ensuring a stable structure.

**Figure 5:**
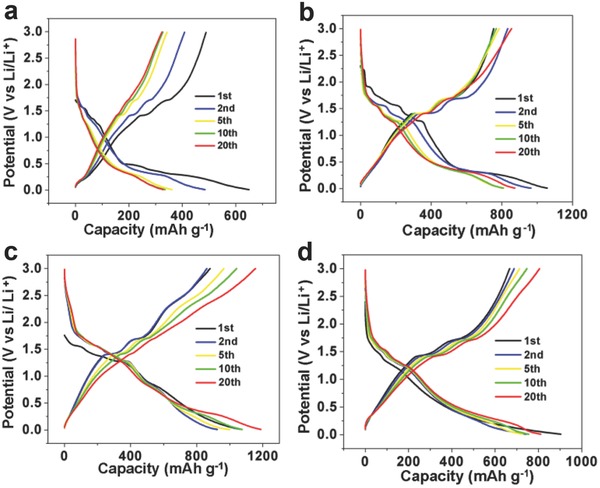
Galvanostatic discharge/charge voltage profiles of a) MoO_2_ particles, b) MoO_2_/GO‐1, c) MoO_2_/GO‐2, and d) MoO_2_/GO‐3 at the current density of 100 mA g^−1^.

**Figure 6:**
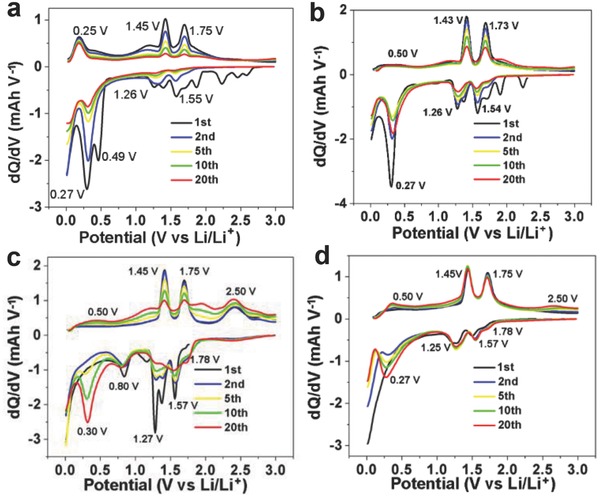
Corresponding differential curves of a) MoO_2_ particles, b) MoO_2_/GO‐1, c) MoO_2_/GO‐2, and d) MoO_2_/GO‐3 at the current density of 100 mA g^−1^.

The electrochemical performance of the GO supported MoO_2_ architectures was tested as anode in half cells, which was investigated by galvanostatic discharge/charge experiments. The MoO_2_ particles synthesized with the identical method but in the absent of the GO supports and the GO were tested as the baselines. The constant current discharge/charge voltage profiles of MoO_2_/GO architecture as well as the MoO_2_ particle electrodes cycled at the current of 100 mA g^−1^ are shown in **Figure** **5**. To get a clearer view, the corresponding differential curves d*Q*/d*V* with the maximum of each peak labeled are provided in **Figure** **6**. The d*Q*/d*V* curves of the pure MoO_2_ particles display two irreversible peaks around 0.27 and 0.49 V at the first cycle (Figure 6a). When the GO was employed as the flexible supports for the MoO_2_/GO architectures, the peak at 0.49 V has disappeared. (Figure 6b–d). The irreversible capacity at the initial cycle is resulted by the decomposition of the electrolyte as well as the formation of solid–electrolyte interphases (SEIs).^32^ These are possibly attributed to that the GO as the flexible supports and backbones making the MoO_2_ more stable. The coulombic efficiency (CE) at the first cycle is one of the vital criterions for the electrochemical energy storage systems.^33^ For Li ion full‐cell testing, the number of Li ions in the system is limited, the irreversible Li ion loss will lead to the capacity loss permanently.^32^ The initial discharge capacities of the pure MoO_2_ particles, micrometer MoO_2_/GO, MoO_2_/GO nanohoneycomb, and layered MoO_2_/GO are 914, 1057, 1075, and 904 mAh g^−1^, respectively. The initial CE of pure MoO_2_ is 75.3%, the corresponding initial CEs of the micrometer MoO_2_/GO, MoO_2_/GO nanohoneycomb and layered MoO_2_/GO are 71.3%, 82.1%, and 74.0%, respectively. The CE of the MoO_2_/GO nanohoneycomb exhibits the highest value among the MoO_2_/GO architectures, which is also higher than the pure MoO_2_ particles, attributed to its unique nanohoneycomb microstructure constructed with ultrafine MoO_2_ fixed by the GO intimately, providing stability of the structure. From the second cycle, the two sets of reversible redox peaks at around 1.55 V (red)/1.75 V (oxid) and 1.26 V (red)/1.45 V (oxid) correspond to insertion of Li into the MoO_2_ to form LiMoO_2_ associated monoclinic–orthorhombic–monoclinic phase transitions with the theoretical capacity 209 mAh g^−1^.^[21,29]^ These are consist with the cyclic voltammetry curves of micrometer MoO_2_/GO, MoO_2_/GO nanohoneycomb, and layered MoO_2_/GO electrodes at a scan rate of 0.1 mV s^−1^ provided in Figure S6 in the Supporting Information. Another set of peaks 0.27 V/0.5 V related to the reversible conversion reaction LiMoO_2_ + 3Li^+^ + 3e^−^ = > Mo + 4Li_2_O induces an additional capacity 627 mAh g^−1^.^20^ As the cycle increase from the 1st to the 2nd, next to the 5th, 10th, and 20th, it is amazing that the peak at 0.25 V of the MoO_2_/GO nanohoneycomb and layered MoO_2_/GO increases during the whole 20 cycles (Figure 6c,d), exhibiting a cycle induced activated process. Theses cycle induced activation process occurred in fine MoO_2_ particles, especially nanoscale MoO_2_.^23^, ^34^, ^35^ While that peak of the pure MoO_2_ particles have a significant decay as cycling. As to the micrometer MoO_2_/GO, it decreases from the first cycle to 10th cycle than increases from the 10th cycle to the 20th cycle, showing a transition between the MoO_2_/GO nanohoneycomb, layered MoO_2_/GO and bulk MoO_2_ particle, possibly attributed to that the size of MoO_2_ of micrometer MoO_2_/GO is in micrometer lever, which is between nanoscale MoO_2_ in the MoO_2_/GO nanohoneycomb and bulk lever MoO_2_. Another peaks at 2.50 V (red)/1.78 V (oxid) is ascribe to GO (The constant current discharge/charge voltage profile and the corresponding d*Q*/d*V* of the GO are provided in Figure S7, Supporting Information). It can be observed that peaks occurred in the MoO_2_/GO nanohoneycomb and layered MoO_2_/GO. This is attributed to that the quantity of GO in the MoO_2_/GO nanohoneycomb and layered MoO_2_/GO is higher than that of micrometer MoO_2_/GO. Especially, as to MoO_2_/GO nanohoneycomb, the ultrafine particle MoO_2_ adjacent on the GO supports, benefitting the GO exposed, providing the possibility for Li ion to access to the GO. The GO in the MoO_2_/GO nanohoneycomb and layered MoO_2_ function as both supports and active materials.

**Figure 7:**
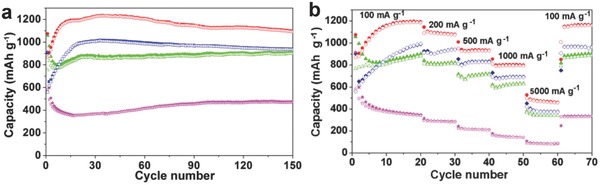
a) Cycle performance and b) rate capacities of MoO_2_ particulates (pink), MoO_2_/GO‐1 (green), MoO_2_/GO‐2 (red), and MoO_2_/GO‐3 (blue).

To show an overall view of the electrochemical performance, the MoO_2_/GO architecture electrodes were tested for 150 cycles at the current density of 100 mA g^‐1^. The pure MoO_2_ particles were tested as the baseline, which is shown in **Figure** **7**a. The capacity of the pure MoO_2_ particle baseline displays a continuous decrease to 356 mAh g^−1^ until the 17th cycle then gradually increase afterward, the capacity reaches to 470 mAh g^−1^ at the 100th cycle, which is consistent with MoO_2_ with the size in microscale.^25^, ^26^ As we have already discussed above, the micrometer MoO_2_/GO also exhibits an induced activation process. The discharge capacity of the micrometer MoO_2_/GO decreases to 801 mAh g^−1^ at the 7th cycle then continuously increases to 901 mAh g^−1^ at the 100th cycle. The MoO_2_/GO nanohoneycomb clearly displays the best cycle performance. The reversible discharge capacity achieves to 1004 mAh g^−1^ at the 4th cycle, with the corresponding CE 98.2%. Then it increases to a peak capacity 1235 mAh g^−1^ at the 34th cycle. Even after 100 cycles the discharge capacity still remains 1127 mAh g^−1^, which is 4.9% higher than the initial capacity. The calculated theoretical capacity (836 mAh g^−1^) is on the basis of phase transitions, and conversion reaction.^23^, ^34^ The substantial additional capacity beyond the theoretical value suggests that all the reversible Li^+^ storage mechanisms are not to be accounted for. Surface adsorption is partially responsible for the extra capacity beyond the theoretical value.^19^, ^35^ Another conceivable explanation for the extra capacity is that the formation of a polymer gel on the surface of nanostructured conversion electrodes during lithiation, with its consequent dissolution upon delithiation.^24^ The layered MoO_2_/GO displays a similar trend as that of MoO_2_/GO nanohoneycomb but the value is around 350 mAh g^−1^ lower than that of MoO_2_/GO nanohoneycomb from the 10th cycle, possibly contributed to its larger size of the pores and particles. When further test to 150 cycles, the retained capacity of micrometer MoO_2_/GO, MoO_2_/GO nanohoneycomb, and layered MoO_2_/GO are 908, 1095, and 937, respectively. The micrometer MoO_2_/GO electrodes still exhibit very stable, while the MoO_2_/GO nanohoneycomb electrodes deliver the highest capacity. The CE is a key factor for the lithium ion battery. The CEs of the MoO_2_/GO architectures are provided in Figure S8 in the Supporting Information. The MoO_2_/GO nanohoneycomb exhibits the best besides at the 1st mentioned above. From the 3rd to 100th cycle, the CE of MoO_2_/GO nanohoneycomb reaches to 97.5% at the 3rd cycle. While the CEs of MoO_2_ particles and micrometer MoO_2_/GO at the 3rd cycle are 89.1% and 90.1%, respectively. The CEs of MoO_2_ particles and micrometer MoO_2_/GO achieved 97.4% and 97.3% at the 15th cycle, which is 12 cycles later than that of MoO_2_/GO nanohoneycomb reached similar value. This is possibly attributed that the size of MoO_2_ particles and micrometer MoO_2_/GO is significantly larger than that of MoO_2_/GO nanohoneycomb, needing more cycle for adjustments and activation. Moreover, it also strongly affect the overall view of the CEs. The average CEs of the MoO_2_ particles, micrometer MoO_2_/GO, MoO_2_/GO nanohoneycomb, and layered MoO_2_/GO are 98.1%, 97.6%, 99.3%, and 99.0%, respectively. The CEs of the GO are provided in Figure S9 in the Supporting Information. The average CE of the GO used as the supports is 95.6%. The MoO_2_/GO nanohoneycomb is able to achieve to the reversible and their average CEs are higher than both of the MoO_2_ particles and GO supports, possibly attributed that to its unique nanohoneycomb structure constructed with ultrafine MoO_2_ fixed on the GO supports.

The rate performance of the micrometer MoO_2_/GO, MoO_2_/GO nanohoneycomb, and layered MoO_2_/GO is investigated to observe their potential for fast charge/discharge, the MoO_2_ particulates were also tested as the baselines (Figure 7b). The rate performance of the GO supports is provided in Figure S10 in the Supporting Information. Generally, the MoO_2_/GO architectures show much higher capacity than the GO and MoO_2_ particulates at all tested current densities. Among them, the MoO_2_/GO nanohoneycomb displays the best. At a current density of 100 mA g^−1^, at the first cycle, the GO shows the discharge capacity as high as 1183 mAh g^−1^, then it decreases very fast. After 20 cycles, the residue capacity is as low as 520 mAh g^−1^, agreeing well to the previous works.^36^ The initial discharge capacities of the micrometer MoO_2_/GO, MoO_2_/GO nanohoneycomb, and layered MoO_2_/GO and the reference MoO_2_ particles are 1057.5, 1074.6, 903.9, and 914.9 mAh g^−1^, respectively. The second discharge capacities of micrometer MoO_2_/GO, MoO_2_/GO nanohoneycomb, and layered MoO_2_/GO decrease to 994.1, 906.6, and 651.4 mAh g^−1^. After around five cycles, then the capacities of the hybrid exhibit increasing trend, these phenomena are similar as ultrafine MoO_2_ nanorods,^35^ the hierarchical MoO_2_ nanoarchitectures^30^ as well as MoO_2_ nanonetworks.^24^ After 20 cycles, the capacities of the micrometer MoO_2_/GO, MoO_2_/GO nanohoneycomb and layered MoO_2_/GO reach to 900.8, 1193.2, and 990.7 mAh g^−1^, respectively. The reference MoO_2_ prepared under similar conditions but without graphene‐oxide deliver a discharge capacity 350.2 mAh g^−1^, which is consistent to the previous results.^24^, ^37^ At the current densities of 200 mA g^−1^ after 30 cycles, the micrometer MoO_2_/GO, MoO_2_/GO nanohoneycomb, and layered MoO_2_/GO, the GO, and the MoO_2_ particulates show the capacities of 822, 1078, 943, 397, and 286 mAh g^−1^, respectively. At the current density of 500 mA g^−1^ after 40 cycles, the capacities of them are 718, 934, 921, 275, and 211 mAh g^−1^, respectively. When the current density reaches to 1000 mA g^−1^, they display the capacities as high as 631, 801, 694, 191, and 143 mAh g^−1^ after 50 cycles, respectively. At the current density of 5000 mA g^−1^, the capacities are 342, 461, 375, 125, and 85 mAh g^−1^, respectively. The MoO_2_/GO nanohoneycomb shows much higher capacity compared with the other two MoO_2_/GO architectures, attributed to that fine MoO_2_ particles (around 50 nm) anchored on GO supports to form a honeycomb‐like network structure with nanoscale pores (around 50 nm). All these provide efficient space for electrolyte transfer, and significantly decrease the diffusion distance of lithium ions and electrons. After 60 cycles, these samples tested at the current density of 100 mA g^−1^ again, the capacities of the MoO_2_/GO nanohoneycomb and layered MoO_2_/GO are 1169 and 963 mAh g^−1^ after 80 cycles, similar as that at the beginning. These attributed to that porosity structures can buffer against the volume change during lithium insertion–desertion, improving the cycling performance.^38^, ^39^ While, the capacity of micrometer MoO_2_/GO is 1073 mAh g^−1^, which is 8.4% higher than that at the beginning after 20 cycles, possibly attributed to that particles of MoO_2_ of micrometer MoO_2_/GO is the largest one among them, needing more charge–discharge cycles for activation. The trends of the MoO_2_/GO nanohoneycomb, and layered MoO_2_/GO are similar, while the micrometer MoO_2_/GO is transition between the MoO_2_ particulates and the other two MoO_2_/GO architectures, possibly attributed to that the micrometer MoO_2_/GO has smaller quantity of GO and larger size MoO_2_ particles.

**Figure 8:**
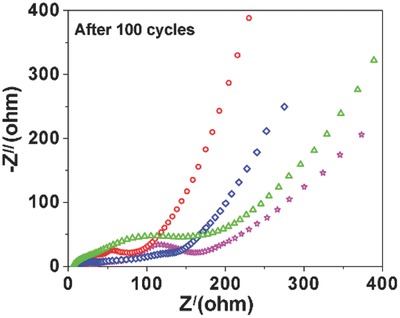
Nyquist plots comparing MoO_2_ particulates (pink), MoO_2_/GO‐1 (green), MoO_2_/GO‐2 (red), and MoO_2_/GO‐3 (blue), after 80 cycles (fully charged).

In order to show the correlation between the electrochemical behavior and the MoO_2_/GO porous structure further, electrochemical impedance spectroscopy (EIS) measurements were carried out in order to compare the impedance differences among the MoO_2_/GO architectures and pure MoO_2_ particulates. The Nyquist plots, shown in **Figure** **8**, were collected from 100 kHz to 0.01 Hz after the electrodes underwent 100 charge/discharge cycles. The equivalent series resistance of the four tested materials is on par due to all of them consist primarily of MoO_2_ with high electrical conductivity. The high‐frequency region of the Nyquist plot also consists of two overlapping semicircles, with their combined diameter being the total charge transfer resistance of the electrodes. In cycled materials the charge transfer resistance is normally attributed to a combination of the charge transfer resistance at the original electrode surface and through the interfaces of the SEI. Among them, the charge transfer resistance in the cycled MoO_2_/GO nanohoneycomb is significantly lower than the micrometer MoO_2_/GO, and layered MoO_2_/GO, as well as the MoO_2_ particulates attributed to a more facile ion transfer pathway at the nanohoneycomb porous structures. Another significant difference comes from the Warburg region, namely the slope of the 45° portion right after the semicircles. The overall length of the Warburg line is indicative of diffusional limitations in the material, with bulk ion insertion and conversion electrodes being solid‐state diffusion limited. The nanohoneycomb porous structures demonstrate a much shorter Warburg region than others, which is expected due to the markedly smaller diffusion lengths in the former.

**Figure 9:**
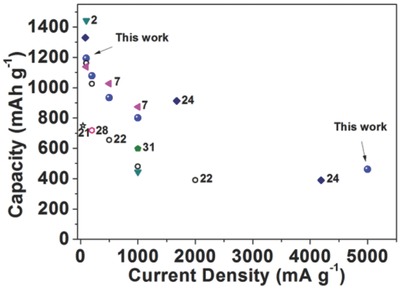
Comparison of the capacity versus current density of the MoO_2_/GO nanohoneycomb (blue balls) versus the state‐of‐the‐art MoO_2_ results from literatures. Hollow, MoO_2_ skeleton porous structures; solid, MoO_2_/carbon‐based composites.

The comparison of our MoO_2_/GO nanohoneycomb with the state‐of‐the‐art in previously literatures on the MoO_2_ base is show in **Figure** **9**. The corresponding data are provided **Table** **2**. Generally at all tested current densities, the carbon supported MoO_2_ composites^3^, ^8^, ^25^, ^27^, ^33^ deliver higher capacities than the MoO_2_ skeleton nanoporous materials at each current density.^23^, ^24^, ^30^ Especially, at a high current density of 1000 mA g^−1^, the capacity of the MoO_2_/N‐GNS,^8^ MoO_2_/GO nanohoneycomb, and MoO_2_/Graphene^34^ are 873.7, 801, and 598 mAh g^−1^, respectively, while the MoO_2_ skeleton porous structure only delivers 480 mAh g^−1^. Even at the current density reach to 4190 mA g^−1^, the MoO_2_/graphene microspheres exhibits a capacity of 390 mAh g^−1^.^26^ As to the MoO_2_/GO nanohoneycomb electrodes, it can deliver a capacity as high as 461 mAh g^−1^ at a much higher current density of 5000 mA g^−1^.

**Table 2:** Cycling performance comparison of MoS_2_/GO nanohoneycomb with state‐of‐the‐art literature for MoO_2_‐based materials, all tested as half cells versus Li/Li^+^

Materials	Synthesis method	Capacity [mAh g^−1^]	Current density [mA g^−1^]	Voltage [V]	Reference
MoO_2_/graphene oxide flexible supports	Preform decomposition	1127 (100) 1078 (typical) 934 (typical) 801 (typical) 461 (typical)	100 200 500 1000 5000	0.01–3.00	This work
Mesoporous crystalline MoO_2_	Template nanocasting strategy	750 (30)	42 (0.05C)	0.01–3.00	Shi et al.^23^
MoS_2_/MoO_2_ nanonetworks	Sulfur‐assisted decomposition	1163 (80) 1025 (typical) 654 (typical) 480 (typical) 390 (typical)	100 200 500 1000 2000	0.01–3.00	Xu et al.^24^
MoO_2_@C nanooctahedrons	Precursor	1442 (50) 443.8 (850)	100 1000	0.01–3.00	Xia et al.^3^
MoO_2_/N‐GNS	Hydrothermal‐calcination method	1138.5 (60) 873.7 (60)	100 1000	0.01–3.00	Wang et al.^8^
MoO_2_/graphene microspheres	Microwave‐assisted hydrothermal process	1330 (100) 913 (typical) 390 (typical)	83.8 (0.1C) 1676 (2C) 4190 (5C)	0.01–3.00	Palanisamy et al.^26^
Hierarchical MoO_2_	Templated	719 (20)	200	0.01–3.00	Sun et al.^30^
MoO_2_/Graphene Nanoarchitectures	Solution and annealed	598 (70)	1000	0.01–3.00	Sun et al.^34^

## 3 Conclusion

In summary, graphene‐oxide‐supported MoO_2_ nanoarchitectures were prepared by decomposing ammonium molybdate/graphene‐oxide preform. The GO acts as a flexible supports to construct MoO_2_ architectures. The inside structures of the architectures can be controlled by altering the percentages of the GO used as flexible supports: micrometer MoO_2_/GO, MoO_2_/GO nanohoneycomb, and layered MoO_2_/GO architectures were achieved at the percentage of GO at 4.3, 15.2, and 20.8 wt%, respectively. The lithium storage performance of the MoO_2_/GO strongly depends on their inside structures. The micrometer MoO_2_/GO, MoO_2_/GO nanohoneycomb, and layered MoO_2_/GO delivered their initial discharge capacities as high as 1057, 1075, and 904 mAh g^−1^ at the current density of 100 mA g^−1^, remaining at the values 901, 1127, and 967 mAh g^−1^ after 100 cycles, respectively. The average coulombic efficiencies of the micrometer MoO_2_/GO, MoO_2_/GO nanohoneycomb, and layered MoO_2_/GO are 97.6%, 99.3%, and 99.0%, respectively. Moreover, they showed the rate capacities 631, 801, and 694 mAh g^−1^ at a higher current density of 1000 mA g^−1^, exhibiting 342, 461, and 375 mAh g^−1^ at the current as high as 5000 mA g^−1^, respectively. The obtained MoO_2_/GO nanohoneycomb exhibits the best, attributed to its honeycomb‐like structure with ultrafine MoO_2_ fixed by the flexible GO supports, as well as the excellent electroconductivity of MoO_2_.

## 4 Experimental Section

*Synthesis of MoO_2_/GO and Reference MoO_2_*: GO was synthesized by using Hummers method.^40^ Graphite flakes were added to the mixture of concentrated H_2_SO_4_ and NaNO_3_, and the mixture was cooled to about 0 °C for 0.5 h. KMnO_4_ was added slowly in the mixture, keeping the temperature below 5 °C for 1 h. The mixture was heated to 35 °C remaining for 2 h. Added water slowly produced a large heat to 98 °C and kept for 2 h. H_2_O_2_ was added to the mixture until the bubbles disappeared. The filtrate was centrifuged (2500 rpm) and the supernatant was decanted away. The remained solid material was washed in succession with 200 mL of water and 200 mL of 5% HCl. The steps were repeated at least three times until the pH was close to 7.0. The product was dried by vacuum freeze drying method.

The MoO_2_/GO was synthesized by decomposition of the prepared ammonium molybdate/GO preform.^28^ In the experiments of the preparation of MoO_2_/GO, 0.36 g GO dispersed in ≈50 mL water under magnetic stirring at room temperature for 48 h. 4.8 g (NH_4_)_6_Mo_7_O_24_·4H_2_O dissolved in ≈50 mL water to form (NH_4_)_6_Mo_7_O_24_ aqueous solution. Then the (NH_4_)_6_Mo_7_O_24_ aqueous solution was added to the GO/H_2_O disperse under magnetic stirring for ≈12 h. After evaporation of H_2_O at ≈120 °C, the ammonium molybdate/GO preform‐1 formed. The preform‐2 and preform‐3 were prepared using 0.36 g GO/3.6 g (NH_4_)_6_Mo_7_O_24_·4H_2_O and 0.36 g GO/1.8 g (NH_4_)_6_Mo_7_O_24_·4H_2_O, respectively.

The MoO_2_/GO‐1, MoO_2_/GO‐2, and MoO_2_/GO‐3 were prepared by decomposition of preform‐1, preform‐2, and preform‐3 at 500 °C for 40 min under the atmosphere of argon, respectively. The as‐prepared products were washed by water for several times and dried under vacuum at 70 °C overnight. Yields of MoO_2_/GO‐1, MoO_2_/GO‐2, and MoO_2_/GO‐3 were 4.53, 3.12, and 1.61 g, respectively.

For the synthesis of the pure MoO_2_ as baseline, MoO_3_ was prepared by decomposition of (NH_4_)_6_Mo_7_O_24_ in air. In brief, 2.00 g (NH_4_)_6_Mo_7_O_24_·4H_2_O was ground. Then it was transferred to a quartz boat. The quartz boat was placed in the middle of the heating zone. The tube furnace was heated to 500 °C with the heating rate of 10 °C min^−1^ and remained at 500 °C for 2 h. Then the system was cooled naturally to room temperature. The as‐prepared product was washed by water for several times and dried under vacuum at 70 °C overnight. Yield: 1.75 g. The reference MoO_2_ was synthesized in a horizontal tube furnace. In brief, 0.50 g of the prepared MoO_3_ was transferred to a quartz boat. The quartz boat was placed in the middle of the heating zone. The tube furnace was heated to 500 °C with a heating rate of 10 °C min^−1^ and remained there for 2 h. The experiments were carried out under the atmosphere of a mixture of hydrogen and argon. (1:1, volume ratio), the flow of the mixture gas was maintained at 10 sccm. Then the system was cooled naturally to room temperature under an argon atmosphere with a flow of 10 sccm. The as‐prepared product was washed by water for several times and dried under vacuum at 70 °C overnight. Yield: 0.40 g.

*Characterization*: XRD analysis of the samples was performed using a Bruker Discover 8 Diffractometer, with Cu *K*_α_ radiation (λ = 1.5406 Å) that was monochromatized using a single Gobel mirror. XPS spectra were recorded on an Axis Ultra spectrometer with Al (Mono) *K*_α_ X‐ray source (1486.6 eV). The base pressure of the analysis chamber was below 10^−9^ mbar.

SEM analysis was performed using a Hitachi S‐4800 field emission scanning electron microscope. The samples were also analyzed using a FIB microscope for micro‐cross‐sectioning. FIB analysis was performed using a Zeiss NVision 40 dual beam system. The FIB used a 30 kV Ga liquid metal ion source with ion currents of 0.15–45 pA. The system was equipped with a vertical SEM column that was situated at 36° relative to the FIB column. Cross‐sections were milled with the FIB and then imaged with the SEM. The FIB SEM was operated at 3 kV to optimize surface sensitivity. TEM analysis was performed using a JEOL 2100 Lab6 TEM, at a 200 kV accelerating voltage. For TEM analysis the samples were dispersed in alcohol with the aid of ultrasonic agitation for several minutes. A drop of the dispersion was deposited onto a copper grid covered by ultrathin carbon film supported by a lacey carbon film. Energy dispersive X‐ray analysis was employed by using a Hitachi S‐3000 N scanning electron microscope. TGA and differential scanning calorimetry analysis (DSC) were performed on an SDT Q600, TA Instruments DSC–TGA, in air with a heating rate of 10 °C min^−1^.

*Electrochemistry*: The electrode materials were prepared by mixing the active material with 15 wt% carbon black and 10 wt% Polyvinylidene fluoride (PVDF) (binder) in *N*‐methylpyrrolidone to form a homogeneous slurry. The well‐mixed slurry was then spread onto a copper foil and dried at 105 °C in a vacuum oven for 12 h. Circular disk electrodes were punched from the foil and used as the anode. The obtained electrodes, polyethene separators, and Li metal foils were assembled into a button cell filed with electrolytes (1 m LiPF_6_ in ethylene carbonate/dimethyl carbonate) in argon atmosphere. Electrochemical characterization was performed in a CR2032‐type coin cell with a multichannel‐current static system (Wuhan, LANHE‐CT2001A, China) in the voltage range of 0.01–3.00 V (vs Li/Li^+^). The discharge/charge experiments were done under a constant current density. Electrochemical impedance spectroscopy (EIS) measurements were carried out using a using a PARSTAT MC electrochemical measurement system (Ametek Co., Ltd. USA). The impedance spectra were recorded on cells in the frequency range between 100 kHz and 10 mHz with a perturbation amplitude of 5 mV.

## Conflict of Interest

The authors declare no conflict of interest.

## Supporting information

Supplementary

## References

[1] A. Ambrosi , Z. Sofer , M. Pumera , Small 2015, 11, 605. 25207749 10.1002/smll.201400401

[2] K. Lu , J. T. Xu , J. T. Zhang , B. Song , H. Y. Ma , ACS Appl. Mater. Inter. 2016, 8, 17402. 10.1021/acsami.6b04587 27322176

[3] G. L. Xia , D. Liu , F. C. Zheng , Y. Yang , J. W. Su , Q. W. Chen , J. Mater. Chem. A 2016, 4, 12434.

[4] K. Tang , X. K. Mu , P. A. van Aken , Y. Yu , J. Maier , Adv. Energy Mater. 2013, 3, 49.

[5] Y. Z. Su , S. Li , D. Q. Wu , F. Zhang , H. W. Liang , P. F. Gao , C. Cheng , X. L. Feng , ACS Nano 2012, 6, 8349. 22931096 10.1021/nn303091t

[6] J. Xiao , X. J. Wang , X. Q. Yang , S. D. Xun , G. Liu , P. K. Koech , J. Liu , J. P. Lemmon , Adv. Funct. Mater. 2011, 21, 2840.

[7] Y. Y. Liang , Y. G. Li , H. L. Wang , J. G. Zhou , J. Wang , T. Regier , H. J. Dai , Nat. Mater. 2011, 10, 780. 21822263 10.1038/nmat3087

[8] X. Wang , Y. Xiao , J. Q. Wang , L. N. Sun , M. H. Cao , J. Power Sources 2015, 274, 142.

[9] X. Gu , J. Yue , L. Chen , S. Liu , H. Y. Xu , J. Yang , Y. T. Qian , X. B. Zhao , J. Mater. Chem. A 2015, 3, 1037.

[10] W. J. Lee , M. H. Park , Y. Wang , J. Y. Lee , J. Cho , Chem. Commun. 2010, 46, 622. 10.1039/b916483a 20062882

[11] L. S. Zhang , L. Y. Jiang , H. J. Yan , W. D. Wang , W. Wang , W. G. Song , Y. G. Guo , L. J. Wan , J. Mater. Chem. 2010, 20, 5462.

[12] Y. G. Li , B. Tan , Y. Y. Wu , Nano Lett. 2008, 8, 265. 18072799 10.1021/nl0725906

[13] P. Poizot , S. Laruelle , S. Grugeon , L. Dupont , J. M. Tarascon , Nature 2000, 407, 496. 11028997 10.1038/35035045

[14] Y. Wu , Y. Wei , J. P. Wang , K. L. Jiang , S. S. Fan , Nano Lett. 2013, 13, 818. 23297784 10.1021/nl3046409

[15] W. M. Zhang , X. L. Wu , J. S. Hu , Y. G. Guo , L. J. Wan , Adv. Funct. Mater. 2008, 18, 3941.

[16] D. Belov , M. H. Yang , J. Solid State Electrochem. 2008, 12, 885.

[17] U. K. Sen , A. Shaligram , S. Mitra , ACS Appl. Mater. Interfaces 2014, 6, 14311. 25062365 10.1021/am503605u

[18] D. B. Rogers , R. D. Shannon , A. W. Sleight , J. L. Gillson , Inorg. Chem. 1969, 8, 841.

[19] L. C. Yang , Q. S. Gao , Y. H. Zhang , Y. Tang , Y. P. Wu , Electrochem. Commun. 2008, 10, 118.

[20] J. J. Auborn , Y. L. Barberio , J. Electrochem. Soc. 1987, 134, 638.

[21] Y. S. Hu , Y. G. Guo , W. Sigle , S. Hore , P. Balaya , J. Maier , Nat. Mater. 2006, 5, 713. 16906142 10.1038/nmat1709

[22] S. Choi , Y. G. Cho , J. Kim , N. S. Choi , H. K. Song , G. X. Wang , S. Park , Small 2017, 13, 1603045. 10.1002/smll.201603045 28098953

[23] Y. F. Shi , B. K. Guo , S. A. Corr , Q. H. Shi , Y. S. Hu , K. R. Heier , L. Q. Chen , R. Seshadri , G. D. Stucky , Nano Lett. 2009, 9, 4215. 19775084 10.1021/nl902423a

[24] Z. W. Xu , H. L. Wang , Z. Li , A. Kohandehghan , J. Ding , J. Chen , K. Cui , D. Mitlin , J. Phys. Chem. C 2014, 118, 18387.

[25] S. Petnikota , K. W. Teo , L. Chen , A. Sim , S. K. Marka , M. V. Reddy , V. V. S. S. Srikanth , S. Adams , B. V. R. Chowdari , ACS Appl. Mater. Inter. 2016, 8, 10884. 10.1021/acsami.6b02049 27057928

[26] K. Palanisamy , Y. Kim , H. Kim , J. M. Kim , W. S. Yoon , J. Power Sources 2015, 275, 351.

[27] X. L. Liu , D. Wu , W. X. Ji , W. H. Hou , J. Mater. Chem. A 2015, 3, 968.

[28] J. F. Huang , Z. W. Xu , L. Y. Cao , Q. L. Zhang , H. B. Ouyang , J. Y. Li , Energy Technol. 2015, 3, 1108.

[29] D. C. Marcano , D. V. Kosynkin , J. M. Berlin , A. Sinitskii , Z. Z. Sun , A. Slesarev , L. B. Alemany , W. Lu , J. M. Tour , ACS Nano 2010, 4, 4806. 20731455 10.1021/nn1006368

[30] Y. M. Sun , X. L. Hu , J. C. Yu , Q. Li , W. Luo , L. X. Yuan , W. X. Zhang , Y. H. Huang , Energy Environ. Sci. 2011, 4, 2870.

[31] K. Chang , W. X. Chen , ACS Nano 2011, 5, 4720. 21574610 10.1021/nn200659w

[32] Q. L. Zhang , X. C. Xiao , W. D. Zhou , Y. T. Cheng , M. W. Verbrugge , Adv. Energy Mater. 2015, 5, 1401398.

[33] X. Q. Zhang , X. N. Li , J. W. Liang , Y. C. Zhu , Y. T. Qian , Small 2016, 12, 2484. 26997521 10.1002/smll.201600043

[34] Y. M. Sun , X. L. Hu , W. Luo , Y. H. Huang , ACS Nano 2011, 5, 7100. 21823572 10.1021/nn201802c

[35] B. K. Guo , X. P. Fang , B. Li , Y. F. Shi , C. Y. Ouyang , Y. S. Hu , Z. X. Wang , G. D. Stucky , L. Q. Chen , Chem. Mater. 2012, 24, 457.

[36] T. Bhardwaj , A. Antic , B. Pavan , V. Barone , B. D. Fahlman , J. Am. Chem. Soc. 2010, 132, 12556. 20731378 10.1021/ja106162f

[37] Y. M. Sun , X. L. Hu , W. Luo , Y. H. Huang , J. Mater. Chem. 2012, 22, 425.

[38] J. S. Chen , T. Zhu , X. H. Yang , H. G. Yang , X. W. Lou , J. Am. Chem. Soc. 2010, 132, 13162. 20822139 10.1021/ja1060438

[39] X. Wang , L. J. Yu , X. L. Wu , F. L. Yuan , Y. G. Guo , Y. Ma , J. N. Yao , J. Phys. Chem. C 2009, 113, 15553.

[40] W. S. Hummers , R. E. Offeman , J. Am. Chem. Soc. 1958, 80, 1339.

