# Supplementary material for: Constructing MoO2 Porous Architectures Using Graphene Oxide Flexible Supports for Lithium Ion Battery Anodes
Source: Glob Chall. 2017 Aug 28;1(7):1700050. doi: 10.1002/gch2.201700050 (PMC6607128; doi:10.1002/gch2.201700050)
Supplement: Supplementary file 1 — Supplementary [file GCH2-1-1700050-s001.pdf]

Copyright WILEY-VCH Verlag GmbH & Co. KGaA, 69469 Weinheim, Germany, 2017.

Global Challenges
Open Access

Supporting Information

for *Global Challenges,* DOI: 10.1002/gch2.201700050

Constructing MoO_2 Porous Architectures Using Graphene Oxide Flexible Supports for Lithium Ion Battery Anodes

Zhanwei Xu, Kai Yao, Hao Fu, Xuetao Shen, Xintong Duan, Liyun Cao, Jianfeng Huang, and Huanlei Wang**

Copyright WILEY-VCH Verlag GmbH & Co. KGaA, 69469 Weinheim, Germany, 2016.

Supporting Information

Constructing MoO_2 porous architectures using graphene oxide flexible supports for lithium ion battery anodes

Zhanwei Xu, [a] *Kai Yao,* [a] *Hao Fu,* [a] *Xuetao Shen,* [a] *Xintong Duan,* [a] *Liyun Cao,* [a] *Jianfeng Huang,* [*a] *Huanlei Wang* [*b]

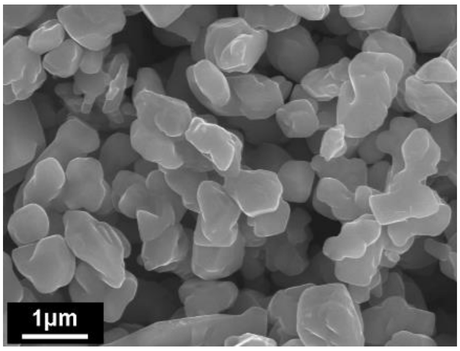

Figure S1. SEM morphology of the MoO_2 particles.

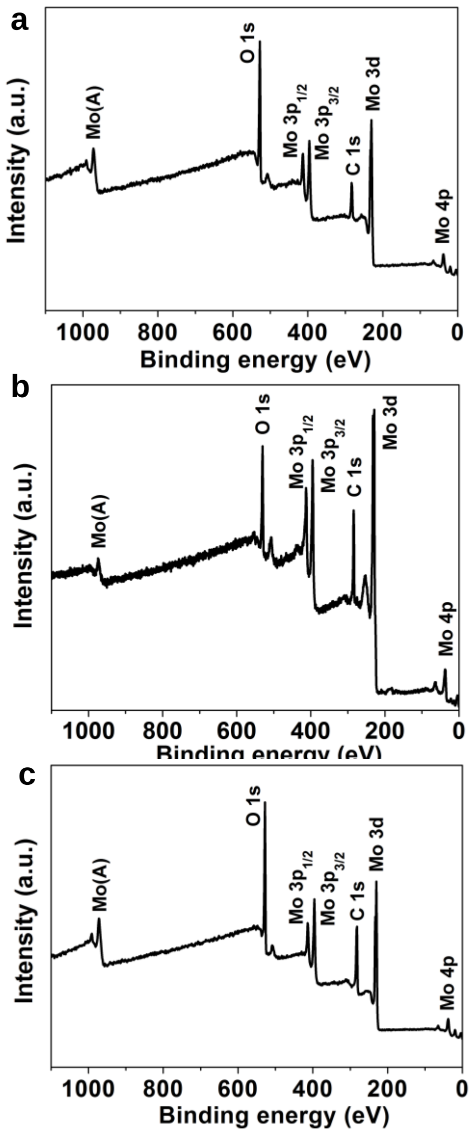

Figure S2. XPS spectra of MoO_2/GO-1, MoO_2/GO-2, MoO_2/GO-3

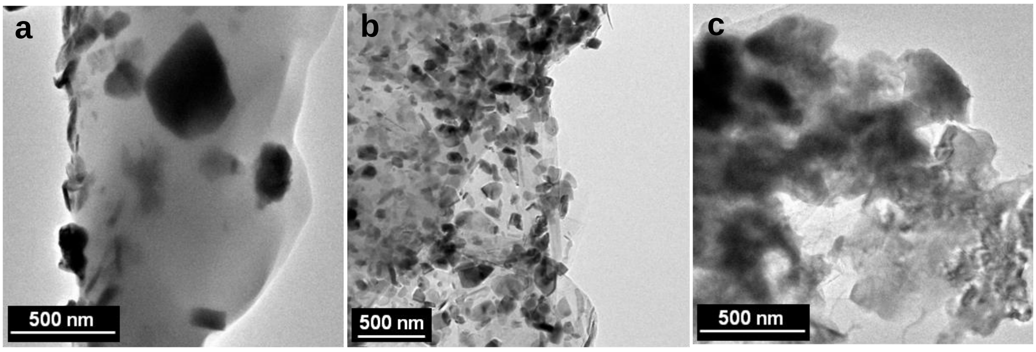

Figure S3. TEM micrographs of (a) MoO_2/GO-1, (b) MoO_2/GO-2, and (c) MoO_2/GO-3.

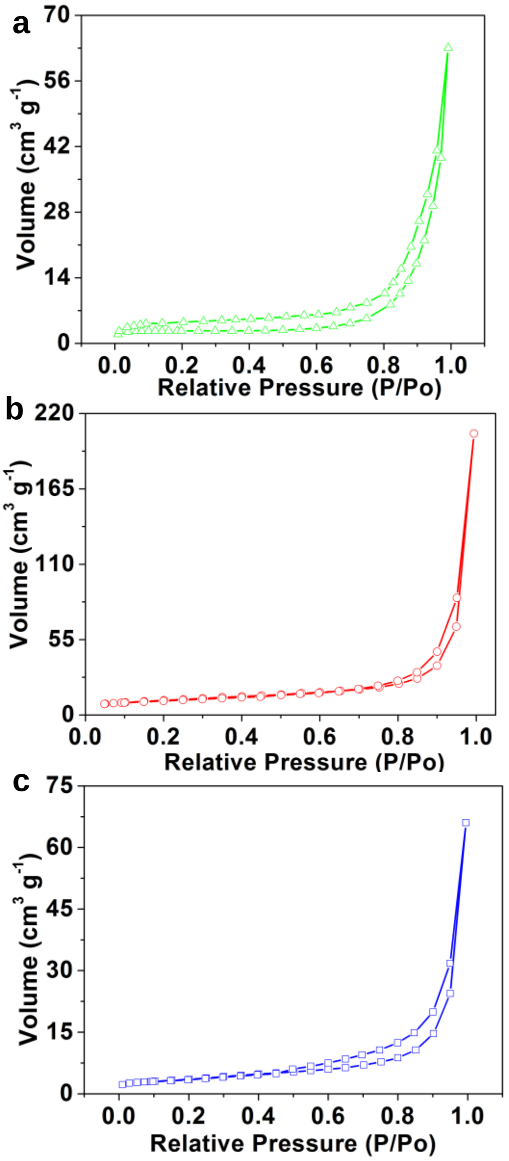

Figure S4. Nitrogen adsorption-desorption isotherms of (a) micron MoO_2/GO, (b) MoO_2/GO nano honeycomb, and (c) layered MoO_2/GO.

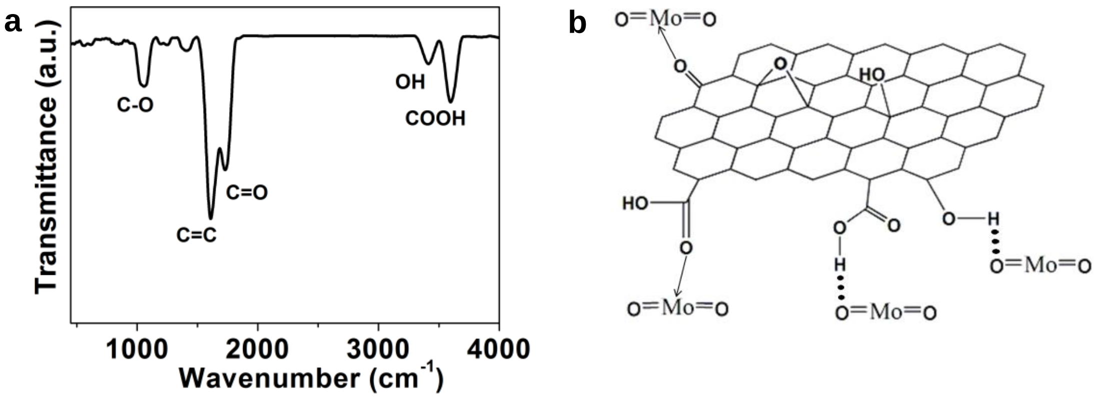

Figure S5. (a) FTIR spectrum of GO. (b) The hydrogen bond and coordination bond between the GO and MoO_2.

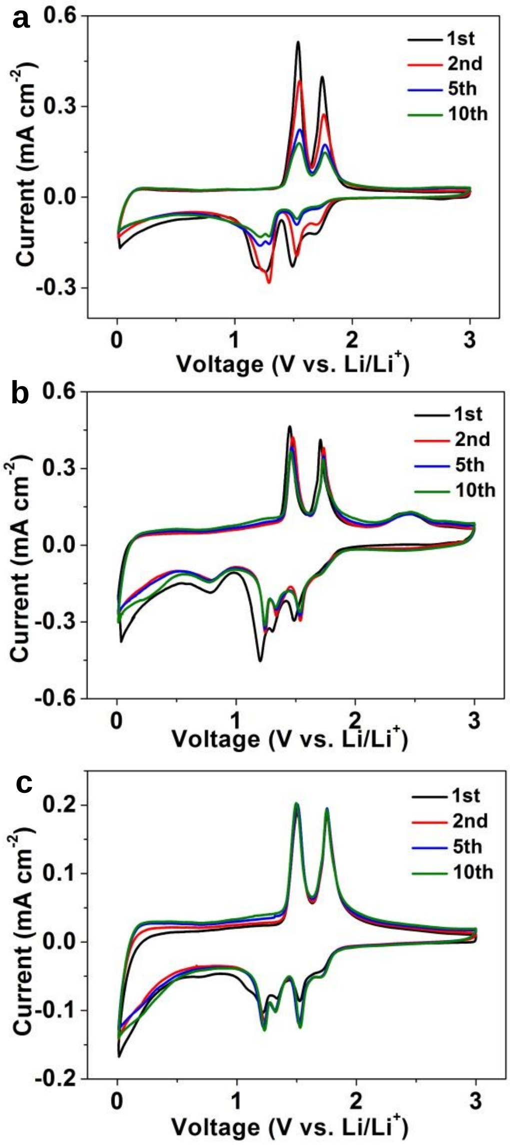

Figure S6. Cyclic voltammetry (CV) curves of (a) MoO_2/GO-1, (b) MoO_2/GO-2, and (c) MoO_2/GO -3 at 0.1 mV s^{-1}.

As shown in Figure S6, the MoO_2/GO composite shows two reversible peaks at 1.55 V (red) / 1.75 V (oxid) and 1.26 V (red)/1.45 V (oxid), which attributed to the reaction of Li into the MoO_2 to form $LiMoO_2$. In addition, as the cycle increase from the 1st to the 2nd, next to the 5th, 10th, the peak at 0.25 V of the MoO_2/GO nano honeycomb, and layered MoO_2/GO increases, exhibiting a cycle induced activated process, which is consist with dQ/dV analysis. From the CV curves, the contributions of non-faradic current are different for the MoO_2/GO materials. The non-faradic current contribution of the MoO_2/GO nano honeycomb are higher than both micron MoO_2/GO and layered MoO_2/GO. These are consist with BET analysis.

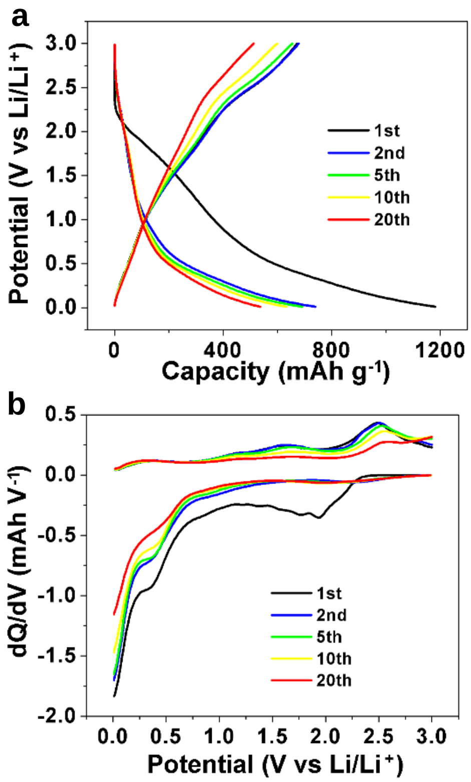

Figure S7. (a) Galvanostatic discharge/charge voltage profiles and (b) the corresponding differential curves of the voltage profiles of the GO at the current density of 100 mA g^{-1}.

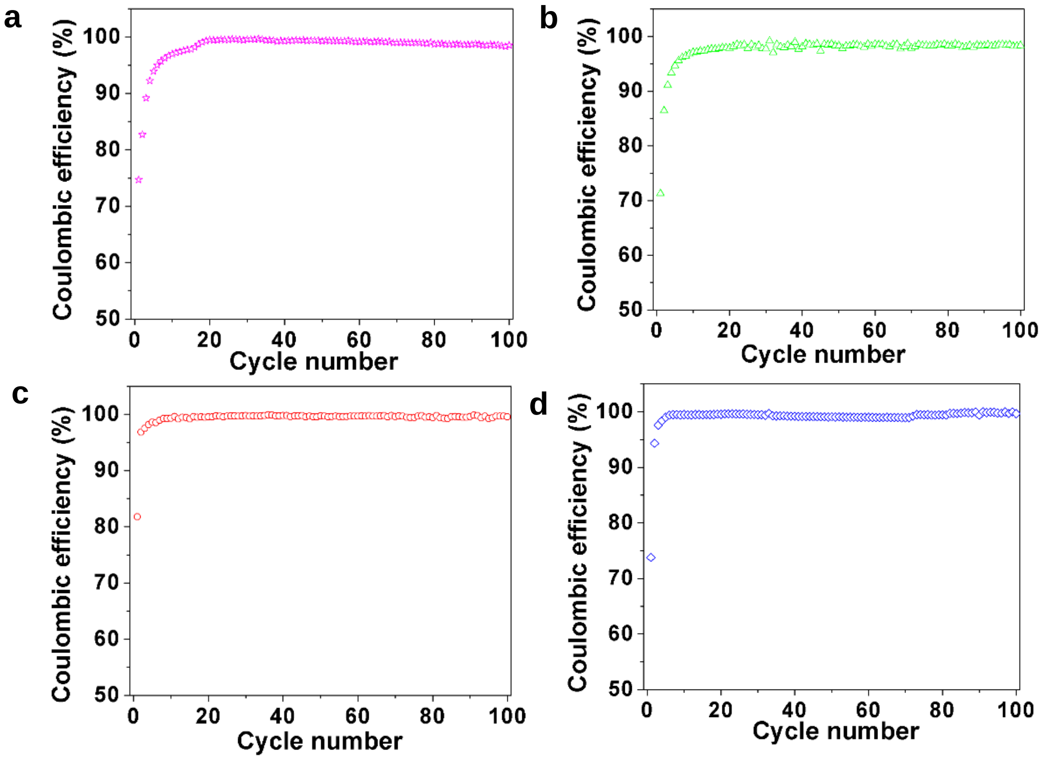

Figure S8. Coulombic efficiency of (a) MoO_2 particles, (b) MoO_2/GO-1, (c) MoO_2/GO-2, and (d) MoO_2/GO -3 at the current density of 100 mA g^{-1}.

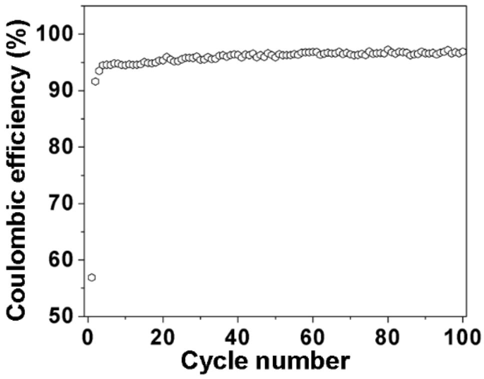

Figure S9. Coulombic efficiency of the GO at the current density of 100 mA g^{-1}.

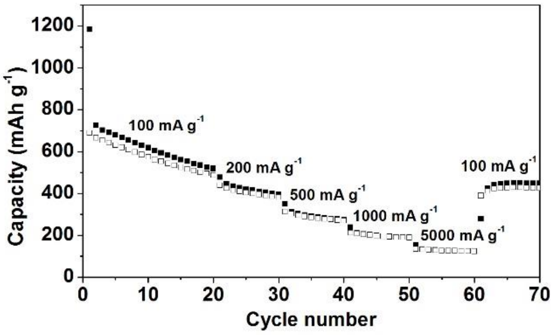

Figure S10. Rate capacities of the GO supports.
